# Simulation of phase contrast angiography for renal arterial models

**DOI:** 10.1186/s12938-018-0471-y

**Published:** 2018-04-16

**Authors:** Artur Klepaczko, Piotr Szczypiński, Michał Strzelecki, Ludomir Stefańczyk

**Affiliations:** 1 Medical Electronics Division, Institute of Electronics, Lodz University of Technology, Łódź, ul. Wólczańska 211/215, 90-924 Lodz, Poland; 2 Department of Diagnostic Imaging, Medical University of Lodz, Łódź, ul. Kopcińskiego 22, 90-153 Lodz, Poland

**Keywords:** Phase contrast angiography, MRI simulation, Blood flow simulation, Vessel segmentation, Kidney vasculature modeling

## Abstract

**Background:**

With the development of versatile magnetic resonance acquisition techniques there arises a need for more advanced imaging simulation tools to enable adequate image appearance prediction, measurement sequence design and testing thereof. Recently, there is a growing interest in phase contrast angiography (PCA) sequence due to the capabilities of blood flow quantification that it offers. Moreover, as it is a non-contrast enhanced protocol, it has become an attractive option in areas, where usage of invasive contrast agents is not indifferent for the imaged tissue. Monitoring of the kidney function is an example of such an application.

**Results:**

We present a computer framework for simulation of the PCA protocol, both conventional and accelerated with echo-planar imaging (EPI) readout, and its application to the numerical models of kidney vasculatures. Eight patient-specific renal arterial trees were reconstructed following vessel segmentation in real computed tomography angiograms. In addition, a synthetic model was designed using a vascular tree growth simulation algorithm. The results embrace a series of synthetic PCA images of the renal arterial trees giving insight into the image formation and quantification of kidney hemodynamics.

**Conclusions:**

The designed simulation framework enables quantification of the PCA measurement error in relation to ground-truth flow velocity data. The mean velocity measurement error for the reconstructed renal arterial trees range from 1.5 to 12.8% of the aliasing velocity value, depending on image resolution and flip angle. No statistically significant difference was observed between measurements obtained using EPI with a number of echos (NETL) = 4 and conventional PCA. In case of higher NETL factors peak velocity values can be underestimated up to 34%.

## Background

### Motivation

Monitoring the state of kidneys functioning becomes an increasingly important task of the modern medical diagnostics. The growing rate of patients with various renal diseases in the developed countries is—besides other factors—linked to the *aging society* phenomenon. Failure in renal operation may impair physiological homeostasis, leading to improper management of electrolytes, acid–base balance perturbation, or deregulation of arterial blood pressure. Kidneys are responsible for blood filtration, removal of water-soluble waste products of metabolism and surplus glucose and other organic substances. The kidney diseases include, among others, acute kidney injury (AKI), chronic kidney disease (CKD) and various cancers (e.g. renal cell carcinoma). The treatment depends on the pathological condition and may require life-long dialysis, kidney removal or transplantation. In any case, exact knowledge of the kidney performance is needed for the proper diagnosis, treatment and follow-up prognosis. Especially in case of CKD, caused by e.g. longstanding hypertension or diabetes mellitus, it is important to continuously and precisely monitor renal function, since early detection of the disease allows prevention of its development to the end stage [1].

One of the diagnostic methods available for assessment of the kidney functioning is phase contrast angiography (PCA)—a non-contrast enhanced magnetic resonance imaging (MRI) protocol. It uses special bipolar gradients implemented within the measurement sequence whose role is to shift MR signal phase relative to blood flow velocity. The imaging sequence is always performed twice, each time with opposite velocity encoding gradients polarity, so that the signal from the stationary tissue is attenuated whereas the signal from the flowing spins is enhanced. Hence, PCA enables visualization of the renal vessel system topology and simultaneously provides with quantitative information about velocity and flow rate of the blood entering the kidney. As such, PCA facilitates measuring of the renal arterial input function and thus opens a way to absolute quantification of the kidney perfusion [2, 3]. With the higher magnetic field strengths, introduction of respiratory gating free breathing protocol, undersampled projection reconstruction [4], and adoption of echo-planar imaging readout [5], phase contrast angiography has emerged as an attractive non-invasive alternative for contrast-enhanced acquisitions. Also, these recent advances have extended applicability of PCA onto the smaller vessels, such as those present in the kidneys.

Clinical usefulness of PCA becomes especially apparent in case of atherosclerotic renal artery stenosis. In order to properly grade the stenosis, not only the level of narrowing but also blood flow velocity must be estimated. The latter information is needed to calculate the acceleration time, i.e. the time between the onset of the cardiac wave and the systolic peak [6]—an important predictor of stenosis. This parameter can be conveniently estimated during the Doppler ultrasound (DUS) examination, which is however inferior to PCA in regard to its ability of reconstructing anatomical details of the arterial system [7]. The intravascular ultrasound can be employed to estimate the level of stenosis or guide the stent placement, but its clinical use is reported mainly in relation to coronary and carotid arteries [8, 9]. Besides, it is considered as an interventional procedure that can performed only by a trained angiographer. Another method which, similarly to PCA, ensures accurate quantification of both arterial geometry and blood velocity is the catheter angiography [10]. Although this technique offers the highest spatial and temporal resolution, it is remarkably invasive, as it uses iodinated contrast agents, requires ionizing radiation, and raises the risk of conscious sedation, bleeding and dissection. These factors exclude catheter angiography as a screening protocol.

Despite the mentioned features, PCA must be used with care. Firstly, MRA tends to overestimate a vessel narrowing due to signal loss caused by turbulent flow regime at locations proximal to stenosis. At distal sections of the artery, vessels diameters can be overestimated, e.g. due to partial volume effects [11].

Secondly, velocity quantification using PCA can also be biased. The measurement errors of the PCA method are linked to several factors and these include: inadequate spatial resolution, oblique orientations of the imaging plane in relation to the velocity encoding direction, phase shifts due to magnetic field inhomogeneity or intra-voxel flow-related offsets, too high or too small value of the aliasing velocity, respiratory motion or aortic pulsation [12]. These impediments can be partly reduced by applying appropriate remedies, such as increased resolution, flow compensation or adjusting the VENC parameter. However, discrepancy between a measured and true value of the flow velocity remains unknown. Its exact assessment in case of real images of biological organs is hampered due to the lack of ground-truth data.

The PCA-based measurements can only be compared with the flow estimates obtained by using some other modality, as showed e.g. in [13], where an attempt was made to validate PCA against a ^133^Xenon washout method. However, it was rather an isolated approach and there is a gap in the literature as far as exact assessment of the PCA-based velocity estimation error is concerned. Besides, the ^133^Xenon washout method is itself error-prone. It measures blood velocity based on microcirculation flow and as such it ignores blood plasma transport from the interstitial to the intravascular space and cannot be treated as a gold-standard. PCA estimations can also be compared to DUS. The latter however measures a range of velocities from a whole volume of interest lying within the field of acquisition of a scanning probe. Thus, their spatial identification cannot be easily resolved.

On the other hand, it must be underlined that knowledge about the scale and source of PCA measurement errors is crucial for concerned planning and possibly optimization of the data acquisition protocol, reliable interpretation of image analysis outcomes, disease diagnosis and its development prognosis. Therefore, an alternative way to estimate the error of PCA, exploited in several studies, though not particularly focused on the kidney, employs computer simulations of MRI [14, 15]. This approach—postulated in this paper—possesses the advantage of the ability to evaluate the impact of individual imaging factors separately. We demonstrate how to efficiently set up the simulation experiment starting from construction of a realistic renal vasculature model. For that purpose we have designed a dedicated computer program called *VesselKnife* and encourage development of custom datasets allowing optimization of imaging protocols for specific clinical applications. To this end we made the code of *VesselKnife* available at [16].

### Related work

The observed progress of MRI simulators indicates that the proposed tools become more and more specialized in modeling specific phenomena underlying image formation process. The very first systems, e.g. [17, 18], based on analytical solution to the Bloch equation, enabled simulation of basic sequences such as T_1_-, T_2_- or proton density-weighted imaging, either using spin- or gradient-echo schemes. Simulators proposed in [19, 20] incorporated, as an additional feature, a possibility to account for magnetic field inhomogeneity induced by differences in imaged objects susceptibility. The authors of [21] focused on functional MRI and thus their solution, called POSSUM, deals with time-dependent characteristics of a virtual organ. It refers to T_1_ and T_2_ constants, as well as to spatial coordinates of an object, which may change either in response to alterations in the blood oxygenation level, or due to respiratory motion. However, in POSSUM movement of virtual spins only affects a resultant image in the form of an artifact but it does not generate signals, as required for MR unenhanced angiography. JEMRIS system, proposed in [22], introduced a numerical solver to update the magnetization vectors state for a modeled spin system. Although such an approach offers a universal simulation scheme, allowing e.g. to account for time-varying gradients, it is computationally more demanding than strategies based on analytical solution. In effect, dynamic applications become less feasible even if high performance computing resources are employed, and there is only a moderate number of published attempts to implement MR angiography sequences using JEMRIS [23]. On the other hand, its advantage—though not linked to numerical approach—is the option to model distribution of magnetic fields (both static and radiofrequency), as well as electric field for multiple transmit and receive coils. Similar capabilities are offered by the software described in [24]. Another step forward is made in MRiLab [25]. In contrast to previous solutions, where each tissue type is modeled as a separate uniform compartment, it proposes a multi-pool exchanging model of interacting spins. The effective usage of this software is, however, constrained by the availability of a CUDA-enabled GPU as an execution platform.

Imaging of the blood flow constitutes a separate research path within the field of MR simulations. A number of papers have been published, where implementations of the Time-of-Flight, black-blood angiography, or PCA protocols are described. From the viewpoint of this study the most relevant works are reported in [14, 15, 26, 27], which are strictly focused on PCA. However, those studies refer to simplistic structures, such as isolated flow channels—straight [14], or with stenosis [27], relatively large vessels with one side branch (e.g. carotid [15] or femoral [26] arteries). Although various flow-related effects linked to the flow regime (pulsatile, turbulent, plug) or imaging technicalities (timing parameters) can be examined with such structures, they do not allow testing of a given measurement sequence with respect to its capabilities in proper reconstruction of a real organ anatomical and functional details.

### Current contribution

In light of the above-presented state of the art in the MRI simulation domain, our contribution consists in providing a solution to two problems simultaneously—simulation of the PCA technique and modeling of the renal arterial system. Specifically, the purpose of this paper was to apply numerical simulations to evaluate capability of the PCA sequences—both conventional and accelerated—to accurately measure the renal hemodynamics and simultaneously reproduce anatomical features of the kidney vasculature. The assumed goal decomposes into two main sub-objectives: (1) construction of digital phantoms of the renal arterial tree, and (2) numerical modeling of the phase contrast angiography protocol. In order to accomplish the first sub-objective we applied two alternative approaches resulting in two categories of vascularity models. The patient-specific phantoms were constructed based on vessel segmentation in high-resolution contrast-enhanced computed tomography (CT) image. Alternatively, a synthetic model was designed using a vessel tree-growth simulation algorithm adapted to the kidney anatomy. Realization of the second sub-objective involved extension of our previously described framework for MR angiography simulation [28] onto the echo-planar imaging (EPI) readout method.

## Methods

### Overview of the methodology workflow

**Fig. 1:**
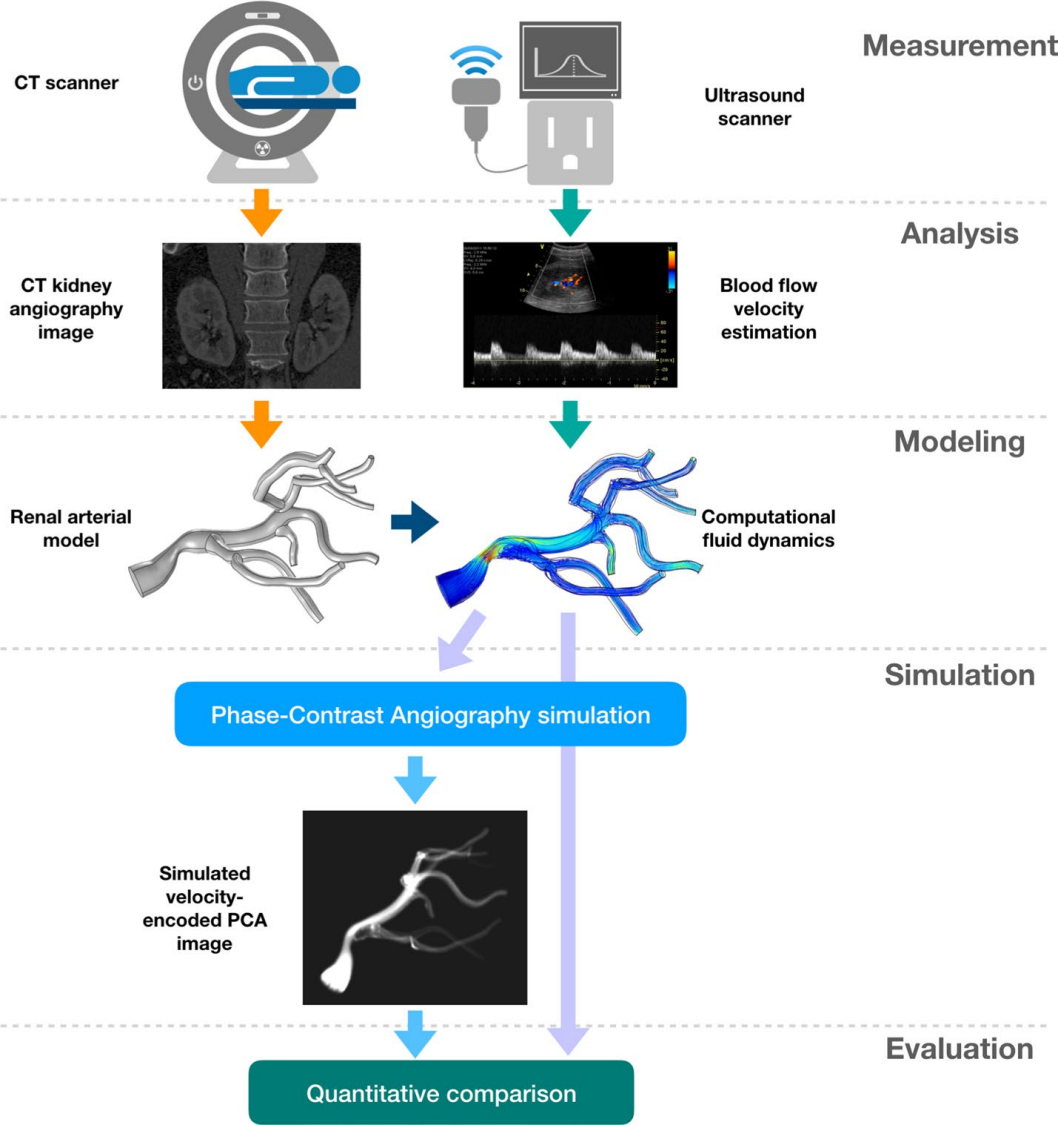
The overview of the experimental workflow implemented in the study

In the part referring to arterial models constructed from real angiography data, the proposed framework can be in general encapsulated into five main steps, as illustrated in Fig. 1. Initially, the images must be collected from a group of patients or healthy volunteers. The data acquisition and its subsequent analysis step embraces two parallel paths: (1) high spatial-resolution scanning in order to achieve detailed information about renal vasculature topology, and (2) functional examination in order to acquire blood flow velocity profiles in the arteries of interest. In our study the data were gathered using CT angiography of the abdomen and Doppler ultrasound. After completion of vessel segmentation and having estimated the blood velocity, the procedure moves on to reconstruction of the arterial tree geometrical model and simulation of blood flow through it. We accomplish both tasks in Comsol Multiphysics [29]—a computational fluid dynamics software. The combined geometrical and functional model is then fed into the magnetic resonance imaging simulator, which implements phase contrast angiography sequence. For that purpose we employ our own solution [28], however each MRI simulator capable of tracking positions of moving virtual spins can be applied. Eventually, the synthesized velocity-encoded phase images are compared with the ground-truth data, i.e. velocity field simulated in Comsol. By playing with the PCA configuration parameters, one gains the knowledge about feasible impact of various imaging factors on the velocity estimation errors. In the following, each of the above-summarized five steps are described in detail and in application to the kidney.

### Real data set acquisition

**Fig. 2:**
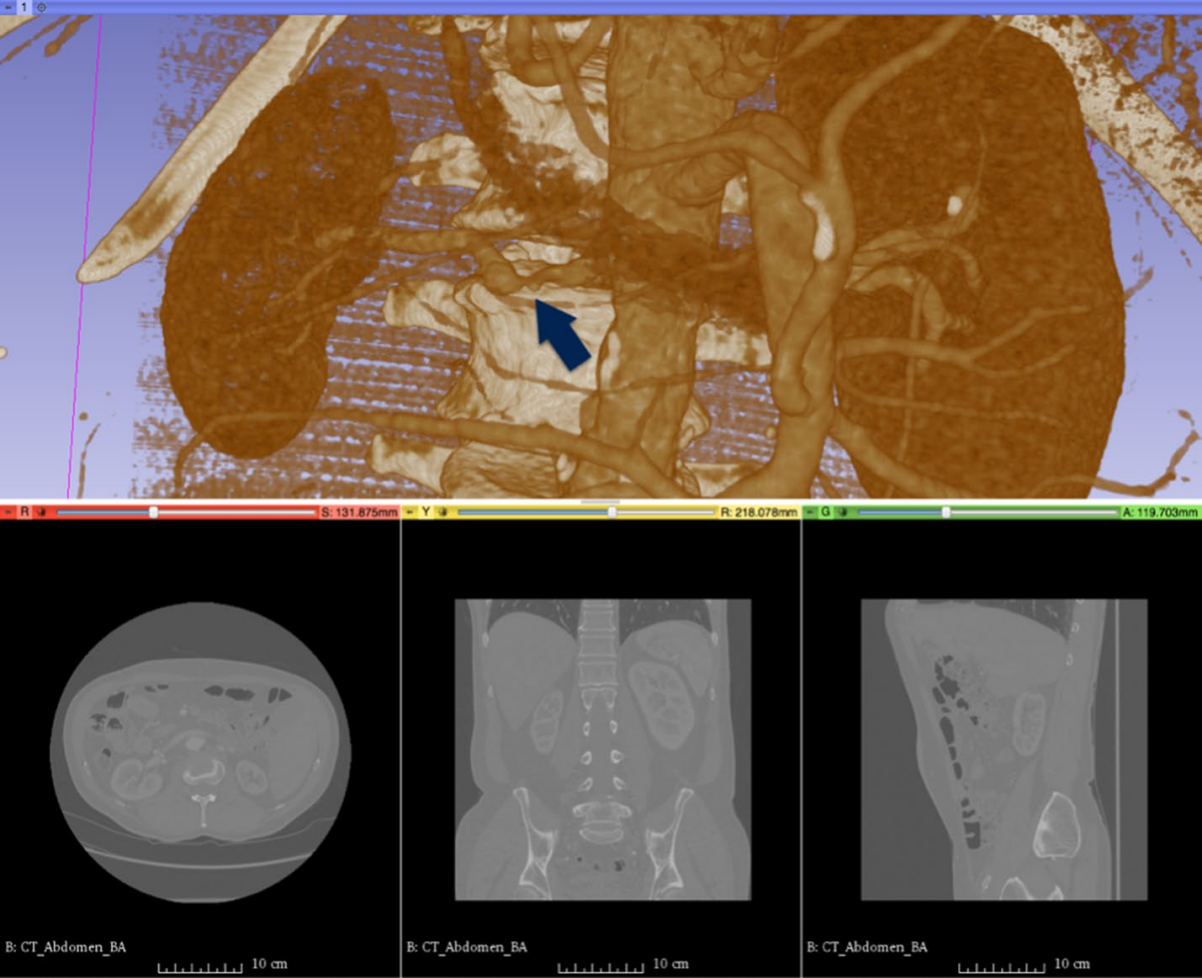
CT data set for a patient with severe stenosis in the left renal artery (blue arrow)—axial, sagittal and coronal slices (bottom row) and volume reconstruction (top panel). Image visualization performed using Slicer 3D software [30]

In our study, the contrast-enhanced angiograms of the abdomen were acquired for four patients. In case of two patients, no abnormal changes in the main renal arteries were observed. In one patient there was a severe renal artery stenosis (RAS) diagnosed in the left kidney (cf. Fig. 2) and one patient had bilateral RAS graded by a specialist as moderate. The data sets were anonymized and processed after collecting a written informed consent from the patients and approval from the local ethics committee (Approval No. RNN/132/17/KE). The in-plane resolution of the images was equal to 0.703 × 0.703 mm^2^ with the matrix size—512 × 512 pixels. The number of axial slices was varied from 571 to 770 (depending on patient) and the spacing between slices, as well as slice thickness was set to 0.625 mm for each individual. Hence, there were overall eight kidney vasculatures reconstructed using real data sets, five of which were normally appearing structures and three degenerated ones referred to as normal or RAS models respectively.

**Fig. 3:**
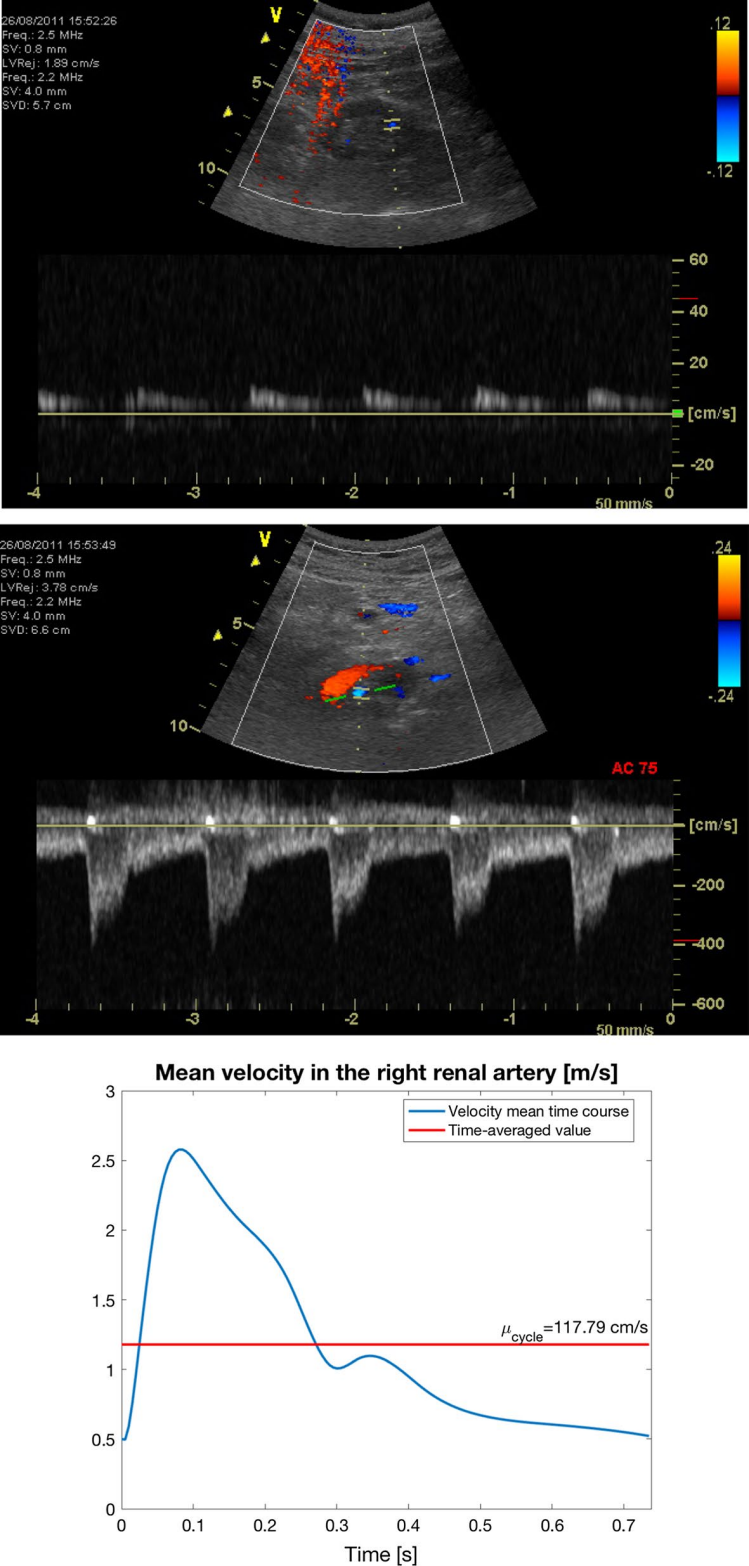
Doppler ultrasound images of the distal vessels in the left kidney (top) and in the right RA (in the middle) acquired for the same patient as the CT data shown in Fig. 2. Bottom: reconstruction of the mean velocity profile in the right RA from the DUS data within one cardiac cycle (blue line). The red horizontal line indicates the calculated velocity value averaged over the cycle duration

In parallel to CT acquisition, the DUS imaging was performed. The collected US images contain information about temporal blood flow velocity in renal artery and distal vessels, i.e. smaller-size vessels located further from the main feeding renal arteries. In the case of one RAS model, the measurement in the left RA was missing. Nevertheless, it was possible to configure blood flow simulation in the reconstructed kidney arterial tree using only the information from the distal vessels. As an example, Fig. 3 presents the US-measured velocity profiles in the right and left kidneys for the patient with severe stenosis.

### Vessel segmentation and vascular tree modeling

Construction of the realistic arterial tree models started from segmentation of vessels in the CT angiogram. This operation was split into two automatic procedures, i.e. vessel enhancement and flood filling. The first step was executed by the help of multi-scale, image Hessian filter with a Gaussian probing kernel [31]. We used five scales to identify voxels potentially belonging to the arterial trees and they were equal to 0.5, 1, 2.5, and 5 mm. The chosen scales range allows detection of most large and medium-sized vessels visible in the image. The filtered image was binarized by performing flood fill operation with the seed points placed at entry regions of the left and right renal arteries. The lower intensity threshold was adjusted as the average brightness of 100 locations randomly sampled from the vessel walls regions and ranged from 128 to 160, again depending on a patient. The upper threshold was set to the top bound of the image dynamic range, as the contrast-enhanced arteries were much brighter than soft tissues. The only structures with a comparable or higher intensity level was the spine and other bones. Unfortunately, the spine orientation usually collides with the abdominal aorta causing its co-segmentation together with the arterial tree during the flood fill operation. Therefore, the spine was manually removed from the segmented objects.

Following segmentation, geometrical description of the vessel system could be extracted. First, the binary objects were skeletonized to determine initial courses of the vessel centerlines. The centerlines defined on the discrete raster were smoothed to obtain continuous descriptions. Smoothing was performed by minimization of the second derivatives calculated at the subsequent centerlines nodes.

For determination of the vessels radii, we employed our algorithm previously described in [32]. In short, the procedure starts from finding the points of intersection between vessel walls and half-lines lead from a given centerline node and equally spaced around that node. Next, a covariance matrix is constructed for the distribution of these points of intersection. The highest eigenvalue of the covariance matrix determines (through its associated eigenvector) the local direction of a vessel, whereas the square root of the smallest one multiplied by an empirically found factor of $\sqrt 2$ gives the radius estimate.

All of the above mentioned automatic procedures, i.e. vessel enhancement, flood-filling, skeletonization and radius estimation were accomplished using the above-mentioned *VesselKnife* software. Partly, the algorithms implemented therein are based on the ITK image processing library [33].

Based on the geometrical description of the kidney vessel tree, its 3-dimensional surface model is constructed. Vessel surface could be determined using visualization libraries (e.g. VTK [34]), and then exported in a 3-D object file format, such as stereolithography (STL). Such an approach is postulated e.g. in [35] in application to cardiac and carotid arteries. Although this mechanism appears to be straightforward and relatively automated, the generated surfaces embrace numerous defects, of which non-manifold edges and self-intersecting faces constitute the most dominant faults that cannot be entirely repaired by surface smoothing during post-processing. In effect, the STL models imported into a computational fluid dynamics (CFD) software may not be tractable as the composition of the volumetric mesh for degenerated surfaces fails.

**Fig. 4:**
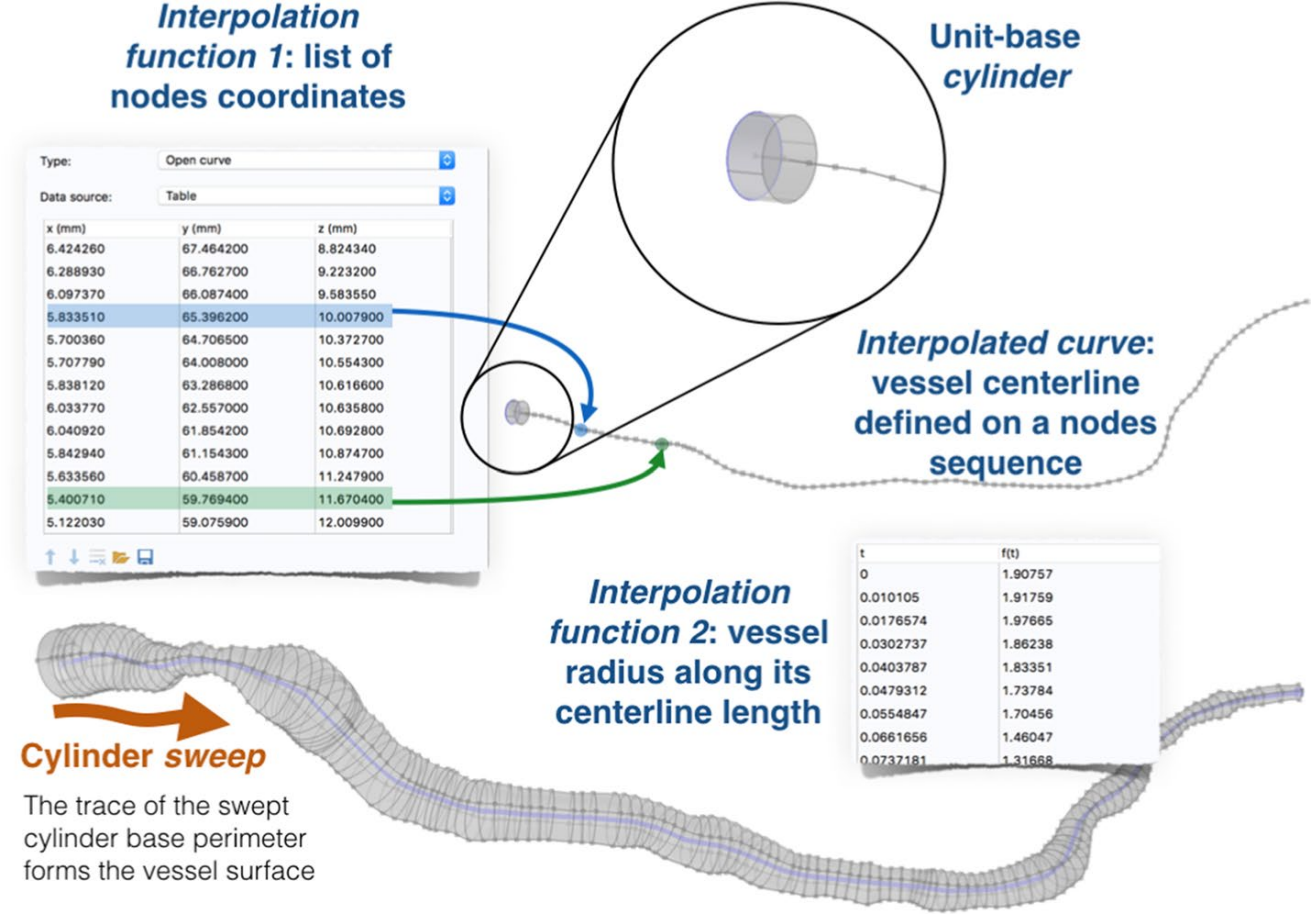
The proposed procedure for construction of a vessel surface in the Comsol Multiphysics software. The swept plane (disk) is a base of a cylinder. Radius of the cylinder base = 1 mm (hence the unit-base cylinder)

Therefore, we used COMSOL built-in tools for 3-D geometrical modeling to reconstruct the renal vasculatures directly from their vector data. Firstly, we imported all centerline courses into the COMSOL’s workspace. Note, a centerline is described by a series of nodes—points in 3-D coordinate space. Then, we represent each centerline as a curve, where subsequent nodes of a centerline become the knots of an interpolated curve (see Fig. 4).

Secondly, each vessel is assigned another interpolation function responsible for the determination of its corresponding vessel radius relative to the distance from this vessel inlet and measured along the centerline. Hence, each centerline length must be first normalized to unity. Subsequent nodes locations are expressed in terms of their relative distance to the starting node. As a result, we obtain a series of pair numbers, which relate the radius of a vessel to its length at discrete locations on a centerline. This relation is eventually interpolated by the cubic spline functions in order to achieve a continuous curve.

Finally, the vessel surface is constructed using the *Sweep* tool of COMSOL. We place a circular (radius = 1 mm) disk (called *plane* in the COMSOL’s environment) at a vessel inlet node and align its normal vector with the local orientation of the vessel centerline. Then, the plane is swept along the centerline. The plane orientation is adjusted according the local vessel direction, and the radius is modified by the appropriate interpolation function. The trace of swept disk perimeter produces a vessel surface. The union of all surfaces forms a kidney arterial tree and can be used in blood flow simulation.

### Synthetic renal vessel tree-growth simulation

For synthesis of the vascular system we adopted the algorithm described in [36, 37]. Tree growth starts with one initial vessel. When a new outlet is selected, a bifurcation is generated. The outlet location is controlled by the shape and spatial position of the perfusion volume and physiological needs of the tissue, expressed in the required flow rate. The bifurcation point is determined in the optimization procedure which is constrained by the following three principles: matter preservation, Poiseuille’s law and the bifurcation law. The first principle states that the blood flow in the bifurcating vessel must be equal to the total flow in the child (outflow) vessels. The second constraint relates the pressure drop along a vessel segment to its associated flow rate, radius, and length, as well as blood viscosity. Eventually, the bifurcation law governs the relation between radii of the parent (inflow) and child vessels. The optimization criterion is the total volume of the bifurcation, which is minimized.

The simulated vessel tree constructed for the purpose of this research was also designed based on a real CT angiogram of the human abdomen. Specifically, we used the CALIX data set from a publically available Osirix database [38] presenting the normal state of the abdominal arteries. However, this time the real image (right kidney) served only as a reference for choosing locations for the synthetic tree terminals. Moreover, the flow rates on the individual outlets of the tree model (hereafter called the CALIX model) were adjusted so that the inlet radius of the root vessel at the end of the optimization process matched the size of the right renal artery visible in the image.

Note, that the output of the tree-growth simulation algorithm is a set of vessel segments modeled as straight tubes. In order to better approximate real vascular systems, the individual vessel segments were redefined to imitate the curved shapes of the vessels and diameter variation along the axes. This remodeling was accomplished in COMSOL, using a similar approach as described for the normal and RAS trees.

### Phase contrast angiography simulation

Our framework for simulation of PCA sequences consists of two integral modules. The first one is responsible for modeling blood flow through the vasculature of an imaged organ. This part is accomplished with COMSOL Multiphysics. The blood flow dynamics is described by the Navier–Stokes equation and solved by assuming a laminar regime, rigid walls, constant viscosity and incompressibility of the fluid. The solution is represented in form of a velocity vector field and flow streamlines, i.e., motion paths of virtual particles traversing the blood vessel system from the inflow to the outflow terminals. This information is then imported by the MRI simulation module.

The import algorithm populates the streamlines along their entire length with particles corresponding to blood spin isochromats. Particles on a streamline are distributed relative to the local blood velocity. If velocity increases, as in a narrowed region, the distances between particles become larger. This strategy ensures the assumed incompressibility of the fluid. Furthermore, the same library keeps track of all moving particles and whenever a given particle passes its trajectory end node, it is replaced by a new one at the inlet. Hence, the vessel tree is constantly filled with particles throughout the whole experiment. Eventually, particle positions can be monitored at arbitrary temporal resolution. Whenever required the simulation time step can be decreased, e.g. during an RF pulse, or enlarged, e.g. during free precession, in order to keep the trade-off between computational precision and complexity. The number of streamlines and the number of particles on a streamline vary for each investigated object and they are adjusted with respect to the resolution of the final simulated image. Our experiments reported in [26] showed that 15–20 particles per 1 mm^3^ are sufficient to reproduce the characteristic effects of the Time-Of-Flight MRA.

In the MRI simulation which follows fluid flow modeling, particles represent portions of the blood and simultaneously carry information on spin isochromats. Their net magnetization state is calculated based on analytical solution to the Bloch equation [39]—a central mathematical formula underlying MR physics. Depending on the stage of the measurement sequence, a magnetization vector $\vec{M}$ of a particle *p* is modified according to an appropriate variant of that solution. For free precession and during gradient encoding steps, $\vec{M}_{p}$ is given by

1 $$ \vec{M}_{p} \left( {t + \delta t} \right) = Rot_{z} \left( {\theta_{g} } \right)Rot_{z} \left( {\theta_{i} } \right)R_{\text{relax}}^{12} \vec{M}_{p} \left( t \right) + R_{\text{relax}}^{1} \vec{M}_{0} , $$

where δ*t* is the simulation time step, *Rot*_*z*_ is a rotation matrix which turns *M*_*p*_ around the *z*-axis either due to the phase encoding gradient ($\theta_{g}$) or field inhomogeneity ($\theta_{i}$). $\vec{M}_{0}$ denotes the equilibrium magnetization. In the designed system it relies solely on proton density. Evolution of a magnetization vector caused by the transverse and longitudinal relaxation phenomena, dependent on tissue-specific *T*_1_ and *T*_2_ constants, is controlled by the matrix $R_{\text{relax}}^{12}$ and the $R_{\text{relax}}^{1}$ vector:

2 $$ R_{\text{relax}}^{12} = \left[ {\begin{array}{*{20}c} {e^{{ - \frac{\delta t}{{T_{2} }}}} } & 0 & 0 \\ 0 & {e^{{ - \frac{\delta t}{{T_{2} }}}} } & 0 \\ 0 & 0 & {e^{{ - \frac{\delta t}{{T_{1} }}}} } \\ \end{array} } \right], $$

3 $$ R_{\text{relax}}^{1} = \left[ {\begin{array}{*{20}c} 0 \\ 0 \\ {1 - e^{{ - \frac{\delta t}{{T_{1} }}}} } \\ \end{array} } \right]. $$

In the excitation phase, we assume that the radio-frequency (RF) pulse duration $\tau_{\text{RF}}$ takes much shorter than the relaxation times. Then, $\vec{M}_{p}$ is flipped from the direction of the main magnetic field by a rotating operator $R_{\text{RF}}$:

4 $$ \vec{M}_{p} \left( {t + \delta t} \right) = R_{\text{RF}} \left( {\alpha_{\text{eff}} } \right)\vec{M}_{p} \left( t \right), $$

where an effective angle $\alpha_{\text{eff}}$ accounts for the off-resonance effects alternating the presumed flip angle $\alpha$ [37]. The RF pulse duration $\tau_{\text{RF}}$ is also divided into time intervals $\delta t$ and spins magnetization vectors are flipped only by a fraction of $\delta t/\tau_{\text{RF}}$.

**Fig. 5:**
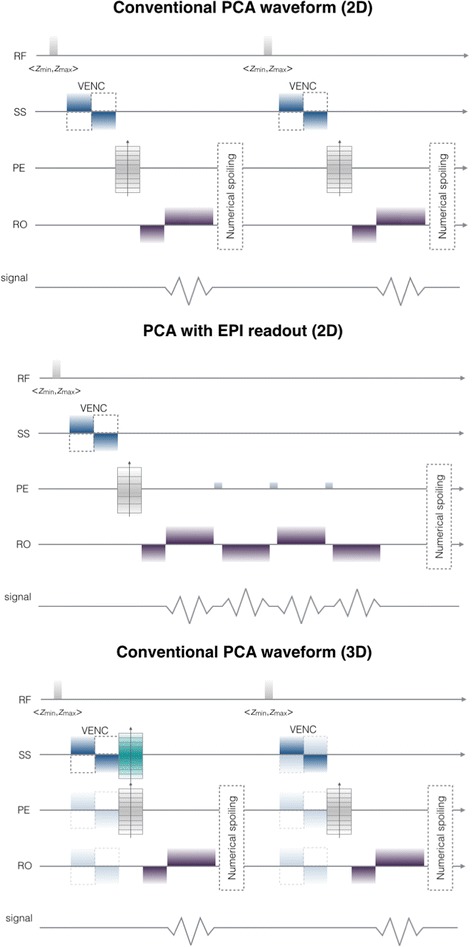
PCA sequence waveforms implemented in the designed MR angiography simulator. In our study, for the 2D PCA velocity encoding gradients are switched on only in the slice-selection (SS) direction. In 3D, velocity encoding is also performed on phase-encoding (PE) and read-out (RO) axes. The dotted lines in the VENC lobes indicate that the sequence is repeated for reversed polarization of the gradients. EPI is presented for NETL = 4 with three gradient blips interleaving consecutive readouts during one TR cycle. Conventional PCA waveforms (2- and 3-dimensional) are presented for two TR cycles

Figure 5 shows sequence waveforms used in our implementation of the phase-contrast angiography. The assumed gradients shape has a rectangular form. The signal acquisition window width *t*_ACQ_ is adjusted to ensure that the Nyquist–Shannon sampling theorem is met. In the conducted experiments, *t*_ACQ_ = 1–2.5 ms, depending on the acquisition protocol (see Table 1). In addition to conventional 2D and 3D PCA, we also designed a 2D echo-planar imaging readout. The latter has been recently adopted for PCA imaging of e.g. pulmonary arteries [40]. Here we study its feasible application to kidney vasculatures as an alternative to other accelerated sequences which employ k-space undersampling or radial acquisitions.

**Table 1:** Acquisition sequence parameters used in the experiments

Parameter name	Vasculature models			
	Normal		RAS	CALIX
Dimension	3D		3D	2D
Readout type	Conventional			Conventional, EPI
TE (ms)	2.5		3	4
TR (ms)	40		40	70
FA	15, 25, 35		20	30
*t*_ACQ_ (ms)	1		1	1.25
In-plane resolution	0.8 × 0.8 mm^2^		0.5 × 0.5 mm^2^ 0.8 × 0.8 mm^2^ 1 × 1 mm^2^	0.5 × 0.5 mm^2^
FOV (mm)	80 × 80		64 × 64	16 × 16
Slice spacing (mm)	1	2.5	1	3
Matrix size	100 × 100		128 × 128 80 × 80 64 × 64	32 × 32
Number of slices	90	36	56	2
VENC (cm/s)	250		100	100, 150
NETL	–		–	4, 8, 16 (EPI)

Moreover, spoiling of the remnant transverse magnetization at the end of each repetition cycle is performed numerically, be zeroing the components of $\vec{M}_{p}$ in the readout (RO) and phase encoding (PE) direction. Also, the RF excitation event is accomplished without explicit gradient lobes in the slice (or slab in 3D) selection (SS) direction. Instead, the simulator selects the particles to excite based on their current position along the *z* axis and prescribed acquisition region (bounded by *z*_min_ and *z*_max_ coordinates).

Note, that the PCA acquisition sequence is always launched twice, with opposite polarization of the velocity encoding gradients. In the post-processing step, based on the two reconstructed phase images we calculate the so-called *phase*-*difference* image $\Delta \varphi$ [41]. Then, velocity in a given *i*-th voxel is estimated as.

5 $$ v_{i} = \frac{\Delta \varphi }{\pi }{\text{VENC}} . $$

### MR image acquisition protocol

In the presented study, the measurement sequences parameters were varied depending on the vasculature model and experimental setting. Details of the acquisition protocols in specific configurations are collected in Table 1.

## Results

In order to show capabilities of the designed simulation framework we arranged a series of experiments which aimed to inspect ability of PCA to properly reproduce the flow velocity and to examine the dependence of PCA-based velocity quantification from various sequence measurement parameters. Therefore, presentation of the results is divided into three parts. Firstly, we show the constructed renal arterial models along with the blood flow simulation setup. Secondly, we study 3D PCA sequence applied to patient-specific arterial trees reconstructed from CT angiograms. Eventually, the conventional 2D readout is compared against echo planar imaging. In each experiment, we refer the PCA measurements to the speed rates determined in COMSOL. The latter values constitute the ground-truth data, whose comparison with the image-derived velocities leads to estimation of the measurement errors associated with specific imaging sequences (Fig. 5).

### Kidney vascular tree models

**Fig. 6:**
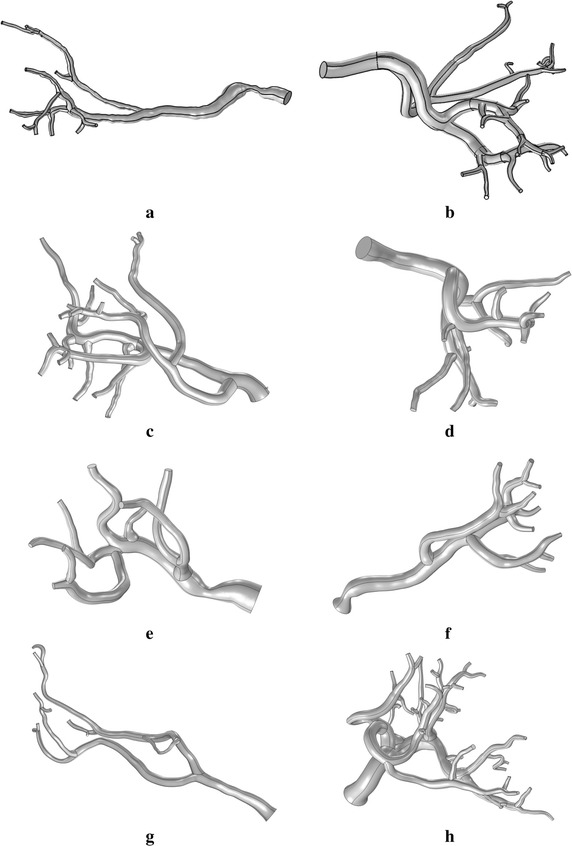
All eight numerical models of kidney vasculatures constructed in the study. Each row correspond to a specific patient. The model in **a** comes from the patient with diagnosed severe stenosis in the left renal artery, whereas models shown in **e** and **f** correspond to the patient with bilateral moderate stenosis. The remaining models, i.e. **b**, **c**, **d**, **g** and **h** correspond to normal vasculatures

Figure 6 presents reconstructed patient-specific models of the kidney vasculatures. The detailed characteristics of the models geometry is summarized in Table 2.

**Table 2:** Summary of geometrical and functional characteristics of the models shown in Fig. 6

Geometrical feature	Model identifier^a^							
	(a)	(b)	(c)	(d)	(e)	(f)	(g)	(h)
RA inlet radius (mm)	3.8	4.7	4.1	3.9	4.5	4.7	2.9	4.2
Average outlet radius (mm)	0.45	0.50	0.78	0.83	0.81	0.76	0.51	0.49
Number of outlets	10	27	16	12	8	9	9	31
Contraction in the RA (%)	80	–	–	–	50	55	–	–
Time averaged mean inlet velocity (cm/s)	10	118	40	25	11	12	20	108

^a^Model identifiers correspond to labeling in Fig. 6

It can be observed, that complexity of the constructed models differ significantly between the objects or even between kidneys from the same object. The smaller number of vessels, especially in the RAS phantoms, is partly due to reduced volume of blood delivered to a given kidney resulting in its decreased performance in blood filtration, contraction of the renal parenchyma, and weaker signal from vessels even in the contrast-enhanced CT angiogram. However, as shown for cases presented in Fig. 6b and h, the proposed methodology allows reconstruction of arbitrarily complex renal vasculatures provided that visibility of the blood vessels in CT images is sufficiently high.

**Fig. 7:**
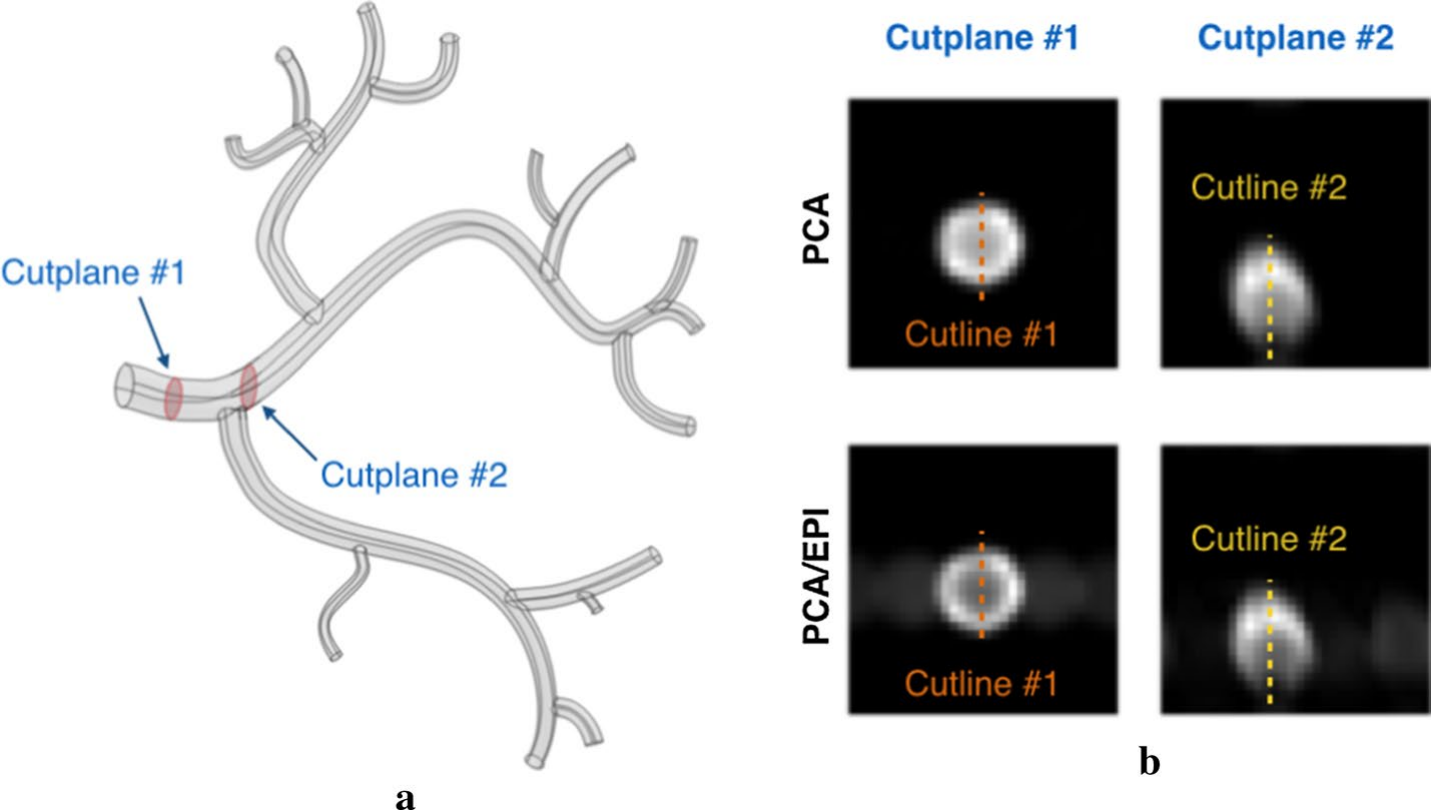
Model of the simulated renal arterial tree (**a**) with the indicated cutplanes and cutlines imaged in the study. Synthesized magnitude images of the cutplane #1 and #2 (**b**) using conventional 2D PCA and EPI-based sequences. Image acquisition parameters: TE/TR/FA = 4 ms/70 ms/30°; in-plane resolution = 0.5 × 0.5 mm^2^; slice width = 3 mm; VENC = 100 cm/s

In case of the simulated tree (Fig. 7a), the number of outlets was set to 14. The diameters of the vessels in this model range from 1.5 mm (minimum outlet) to 5 mm (inlet). This model was used in a 2D experiment with objective to measure the blood velocity in the main renal artery. In relation to kidney location in the original CALIX dataset, the tree was rotated, so that the main feeding renal artery co-aligned with the *Z* axis. This operation has significantly simplified the imaging simulation and the post-processing stage. 2-dimensional PCA gives the most credible velocity estimates when the through-plane flow is maximized. Thus, without rotation, acquired cross-sections of the renal artery must have been selected in configuration with oblique slice orientation. Note, that current implementation of the used angiography simulator disallows oblique slices. Hence, by rotating the tree we made the dominant flow direction coherent with the simulator’s slice-selection axis and it became possible to acquire the designated slices simply in the axial direction.

The blood flow simulation was configured by forcing laminar inflow on the renal arteries inlet boundaries with prescribed average flow velocities. These average velocity values selected for the simulation in the patient-specific models were determined based on the corresponding DUS measurements. In case of the normal tree models we used the time-averaged mean velocities measured in the renal artery (see Table 2 and Fig. 3 as an example). For the RAS model, where the velocity profile in the renal artery was unavailable, we adjusted the inflow velocity to achieve the time-averaged mean value measured for the kidney distal vessels. The experimentally found value was $v_{AVG} = 10 \;{\text{cm/s}}$. Eventually, in case of the CALIX phantom, the flow was forced by setting the pressure difference between the inlet and outlet branches. The inlet pressure was varied to achieve ten different velocity measurement levels. This range was determined in relation to the maximum velocity in the renal vasculature volume. Based on the data reported in the literature for healthy kidneys [42] the presumed velocity interval embraced values from 45 to 93 cm/s.

The presented configuration of the blood flow simulation experiments assume a simplified model of the true renal hemodynamics. One such assumption refers to the blood material, which was treated as homogeneous, Newtonian and incompressible fluid. Moreover, the vessel walls were rigid and stationary. Such an arrangement may be inappropriate even in case of larger arteries of the vascular system, as comprehensively discussed in e.g. [43]. However, despite these simplifications and the previously described constraints linked to MR imaging procedure itself, the reconstructed images exhibit characteristic features of the true angiography, as we qualitatively and quantitatively showed in [28].

## 3D PCA imaging

**Fig. 8:**
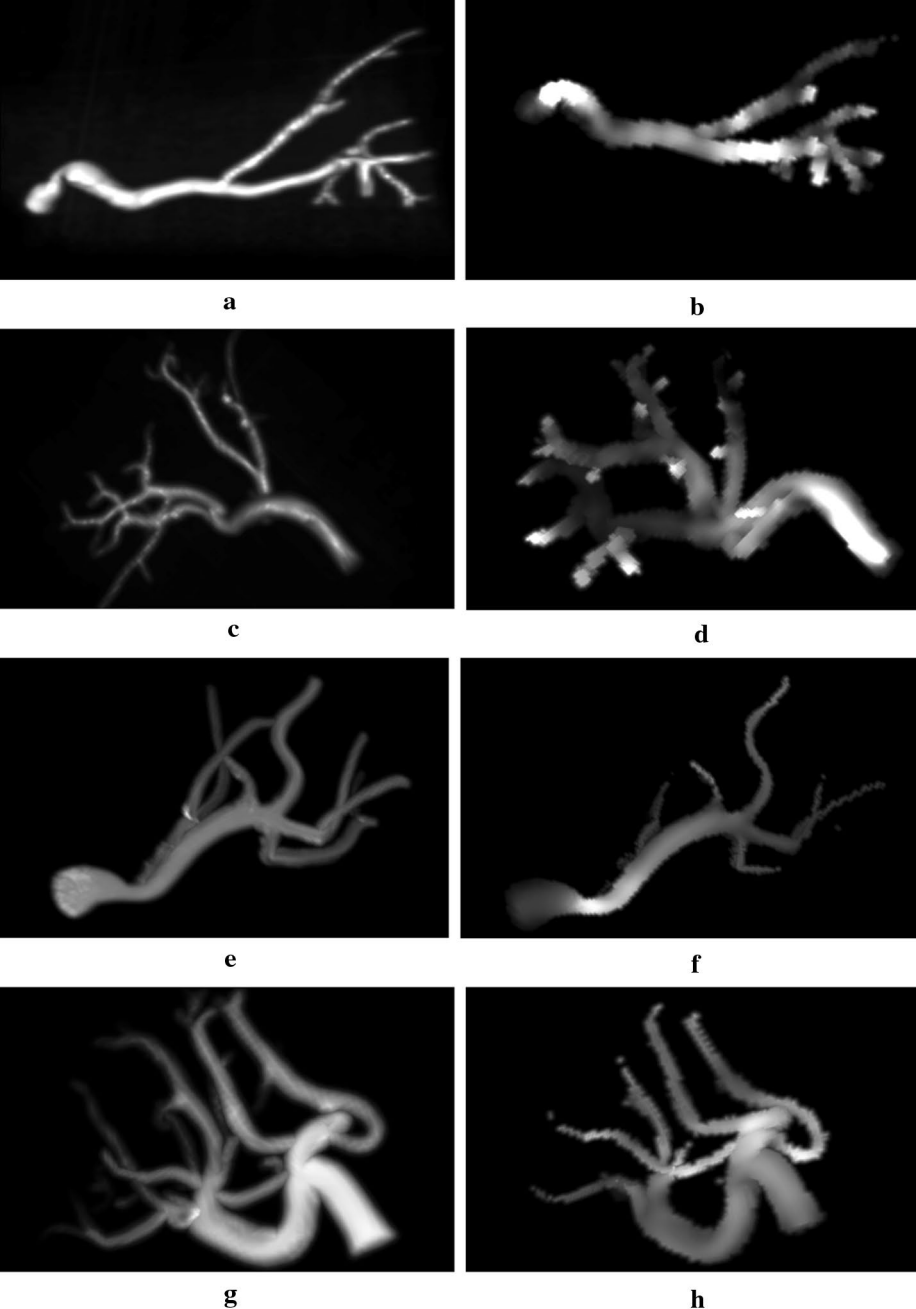
Maximum intensity projections of the 3D magnitude (left column) and velocity-encoded phase (right column) images simulated for models shown in Fig. 6a, b, e and h (from top to bottom)

3D PCA images of the normal and RAS model were simulated for a variety of acquisition parameters. In any case, the velocity encoding gradients were sequentially switched on to obtain images sensitive to velocity in every spatial direction. Hence, for a given acquisition parameters set, we synthesized three separate data sets, from which we reconstructed pairs of magnitude and phase difference images. A magnitude image was used as a mask to cancel out random phase values in locations outside arteries in a corresponding phase image. Then, we calculated maps of velocity magnitude values by evaluating voxel-wise the formula

6 $$ m_{i} = \sqrt {u_{i}^{2} + v_{i}^{2} + w_{i}^{2} } , $$

where *u*, *v*, *w* denote velocity components in *x*, *y*, and *z* direction respectively of an *i*-th voxel in a corresponding (masked) phase difference image processed with Eq. (5). Examples of the simulated magnitude images and reconstructed velocity maps (maximum intensity projections) are shown in Fig. 8a–h.

In order to validate the performed measurements we compared the image-derived velocity magnitude values with the reference data simulated in the flow simulation software. The comparison procedure consisted in finding *K* nearest mesh elements of the model lattice processed in Comsol for every voxel belonging to an arterial tree in the reconstructed velocity map. In our experiments, we tested *K* = 5 and 10. Note, that velocity vector field—as it results from flow simulation—is determined on a lattice composed of approximately 75 × 10^3^ up to 125 × 10^3^ nodes. Thus, one voxel in a simulated PCA image roughly corresponds to 13–20 velocity vectors depending on the acquisition matrix size and slice spacing. The values measured by a PCA sequence in a given voxel is the average of velocities contributed by a subset of particles traversing this voxel region. Location of the particle streamlines is determined in Comsol based on the mesh elements, however their spacing is larger than the mesh resolution. Therefore we calculate the measurement error committed in an *i*-th voxel using two estimates defined as:

7 $$ \bar{e}_{i} = \frac{1}{K}\mathop \sum \limits_{j = 1}^{K} m_{i} - m_{j} , $$

8 $$ \hat{e}_{i} = \min_{j = 1..K} m_{i} - m_{j} , $$

where *m* denotes velocity magnitude. Equation (7) defines the error as the average difference of the measured velocity and the reference velocities determined in the *K* closest mesh elements of a given voxel, whereas (8) finds the minimum of such differences. While the prior estimate appears to be a natural choice, the second one can be justified by the fact, that e.g. in case of the smaller branches (such as the outlet segments) the distribution of the streamlines is more sparse and *K* closest mesh elements may embrace quite different velocity values, which would fictitiously increase the overall error estimate.

**Fig. 9:**
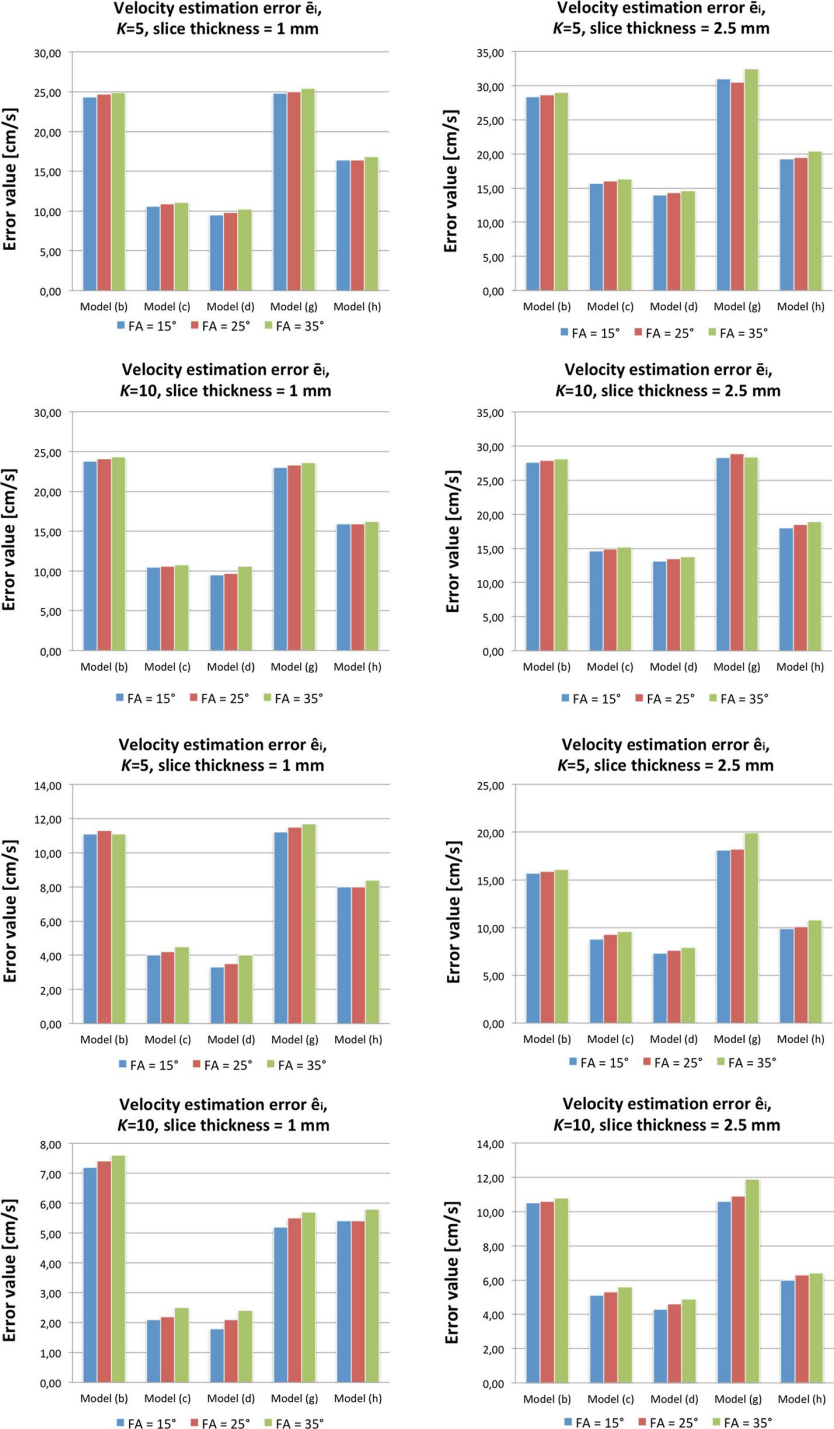
Velocity measurements error estimated based on normally-appearing models

Table 3 collects the measurement errors calculated for various image acquisition parameters for the RAS models. The error estimates obtained for the normal models are graphically visualized in Fig. 9. The reported errors are the mean values averaged over the whole vasculature volumes. As it can be observed, in case of the RAS models the mean velocity measurement error reaches the value of 10.6 cm/s [model (a), in-plane resolution = 1.0 × 1.0 mm^2^]. It is equivalent to 10.5% of the VENC parameter, i.e. the maximum assumed blood velocity. In case of the normal models, the largest error equals to 32.5 cm/s or 12.8% of the VENC value [model (h), flip angle = 25°, slice thickness = 2.5 mm]. On the other extreme, the lowest error estimates are obtained for *K *= 10, where there are more chances to find mesh elements which represent similar speed rates as their corresponding image voxels. For example, in case of the model (d) and the most optimal setting (FA = 15°), $\hat{e}_{i} = 1.8$ cm/s, which falls below 1% of the VENC parameter. The same estimate for the RAS model (f) equals 1.4 cm/s (~ 1.5% of VENC).

**Table 3:** The mean (± standard deviation) of measurement errors committed by the 3D PCA sequence for the RAS models with respect to the in-plane resolution (cm/s)

K	Error estimate	In-plane resolution (mm^2^)								
		0.5 × 0.5			0.8 × 0.8			1.0 × 1.0		
		Model			Model			Model		
		(a)	(e)	(f)	(a)	(e)	(f)	(a)	(e)	(f)
5	$\bar{e}_{i}$	8.6 ± 6.9	8.5 ± 8.0	8.1 ± 5.7	9.7 ± 7.3	9.8 ± 8.3	6.9 ± 5.0	10.6 ± 7.7	10.4 ± 8.4	7.7 ± 5.2
	$\hat{e}_{i}$	4.6 ± 5.9	3.0 ± 4.0	2.6 ± 2.9	5.8 ± 6.4	3.9 ± 4.8	2.5 ± 2.6	6.9 ± 7.0	4.4 ± 5.0	3.2 ± 3.2
10	$\bar{e}_{i}$	8.4 ± 6.5	8.6 ± 7.5	8.3 ± 5.0	9.3 ± 6.8	9.5 ± 7.2	6.9 ± 4.4	10.0 ± 7.2	10.0 ± 7.2	7.4 ± 4.4
	$\hat{e}_{i}$	3.0 ± 4.9	1.6 ± 2.5	1.4 ± 1.8	3.9 ± 5.5	2.0 ± 2.7	1.4 ± 1.6	4.8 ± 6.0	2.2 ± 2.9	1.8 ± 2.1

Model identifiers correspond to labeling in Fig. 6

The best scores are achieved for the finest in-plane resolution and slice-spacing. For example, in case of the RAS phantom (a) and *K *= 10, doubling the matrix size from 64 × 64 to 128 × 128 reduces the average error $\bar{e}_{i}$ from 10 to 8.4 cm/s, whereas the minimum bias $\hat{e}_{i}$ drops on average from 4.8 to 3 cm/s. It means that although improvement of the measurement accuracy is noticeable, it is not as high as the relative increase of the image resolution and in specific applications the gain in estimation precision may not compensate the longer scan time. Eventually, based on the experiment with the normally-appearing models it can be observed that there is small but significant (confidence level = 95%) dependence of the measurement errors from the flip angle. The *t* test performed for the differences of errors calculated between all scores obtained for FA = 25° and 15° and between FA = 35° and 25° results in *p* = 0.0148. The lowest errors are ensured by the smallest FA. Apparently, higher values of flip angle cause the spins residing within the imaging slab for a couple of TR cycles to get saturated and decrease their contribution to the measured signal. In effect, the reconstructed velocity maps posses incomplete information about the blood flow rates.

## 2D conventional PCA vs. EPI imaging

**Fig. 10:**
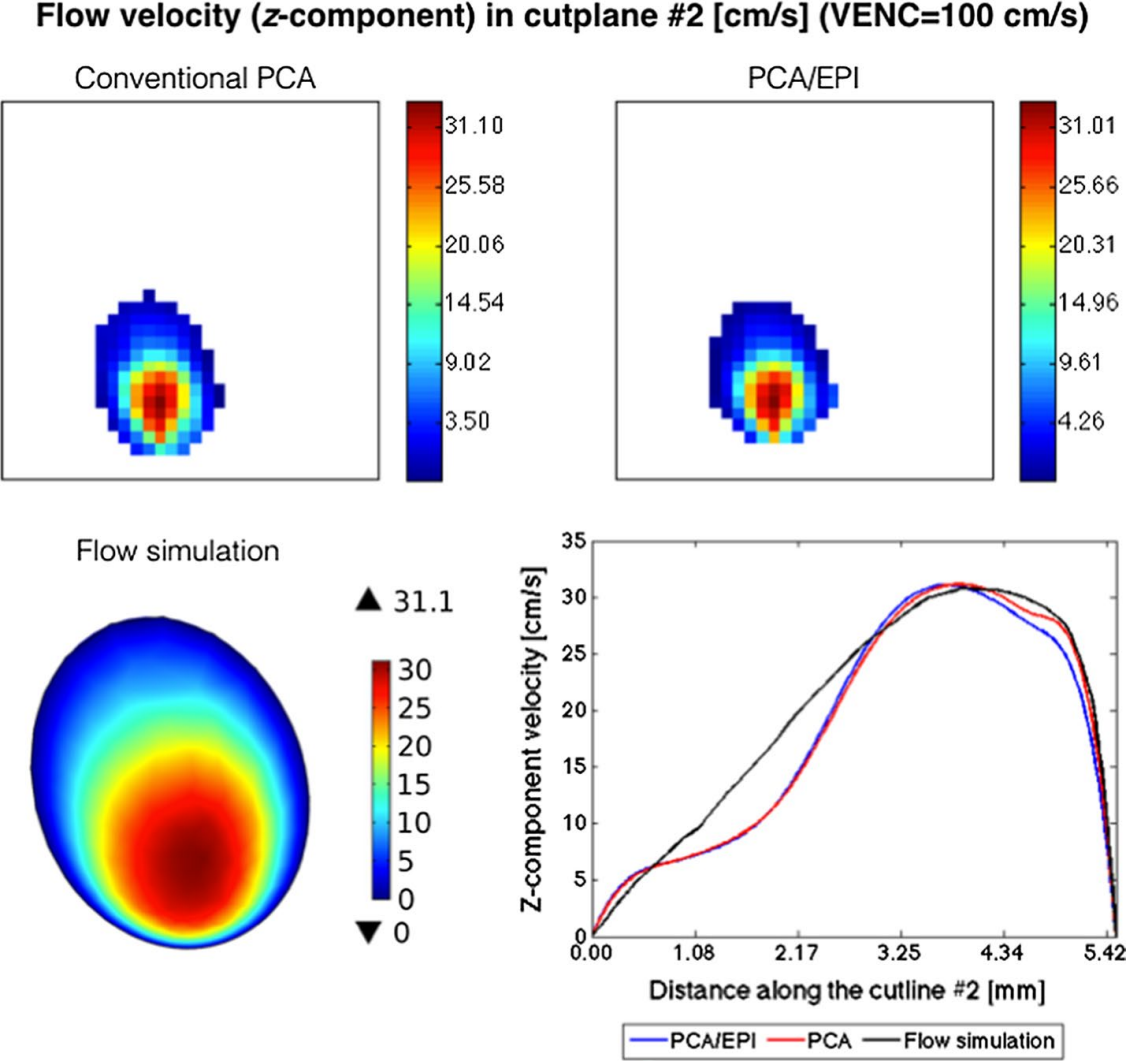
*Z*-component velocity maps reconstructed from conventional PCA and EPI-based images (upper panel). Ground-truth reference map as simulated in Comsol Multiphysics is shown at the bottom-left. Velocity profiles along the cutline #2 (bottom-right) exhibit high-level of correspondence

**Fig. 11:**
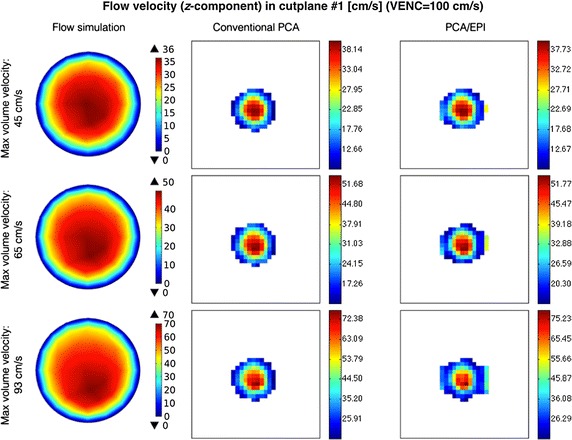
Distribution of *z*-component flow velocity through cutplane #1 of the CALIX model. Left column: plots obtained in the flow simulation software. Middle and right columns: color-coded velocity maps obtained by the designed MR-PCA simulator using conventional and echo-planar imaging readouts respectively (VENC = 100 cm/s)

**Fig. 12:**
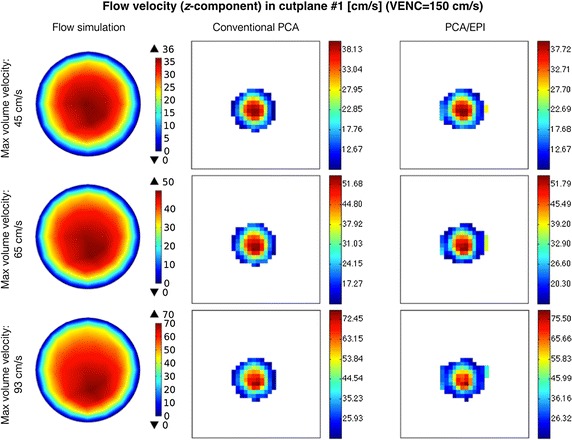
Distribution of *z*-component flow velocity through cutplane #1 of the CALIX model. Left column: plots obtained in the flow simulation software. Middle and right columns: color-coded velocity maps obtained by the designed MR-PCA simulator using conventional and echo-planar imaging readouts respectively (VENC = 150 cm/s)

**Fig. 13:**
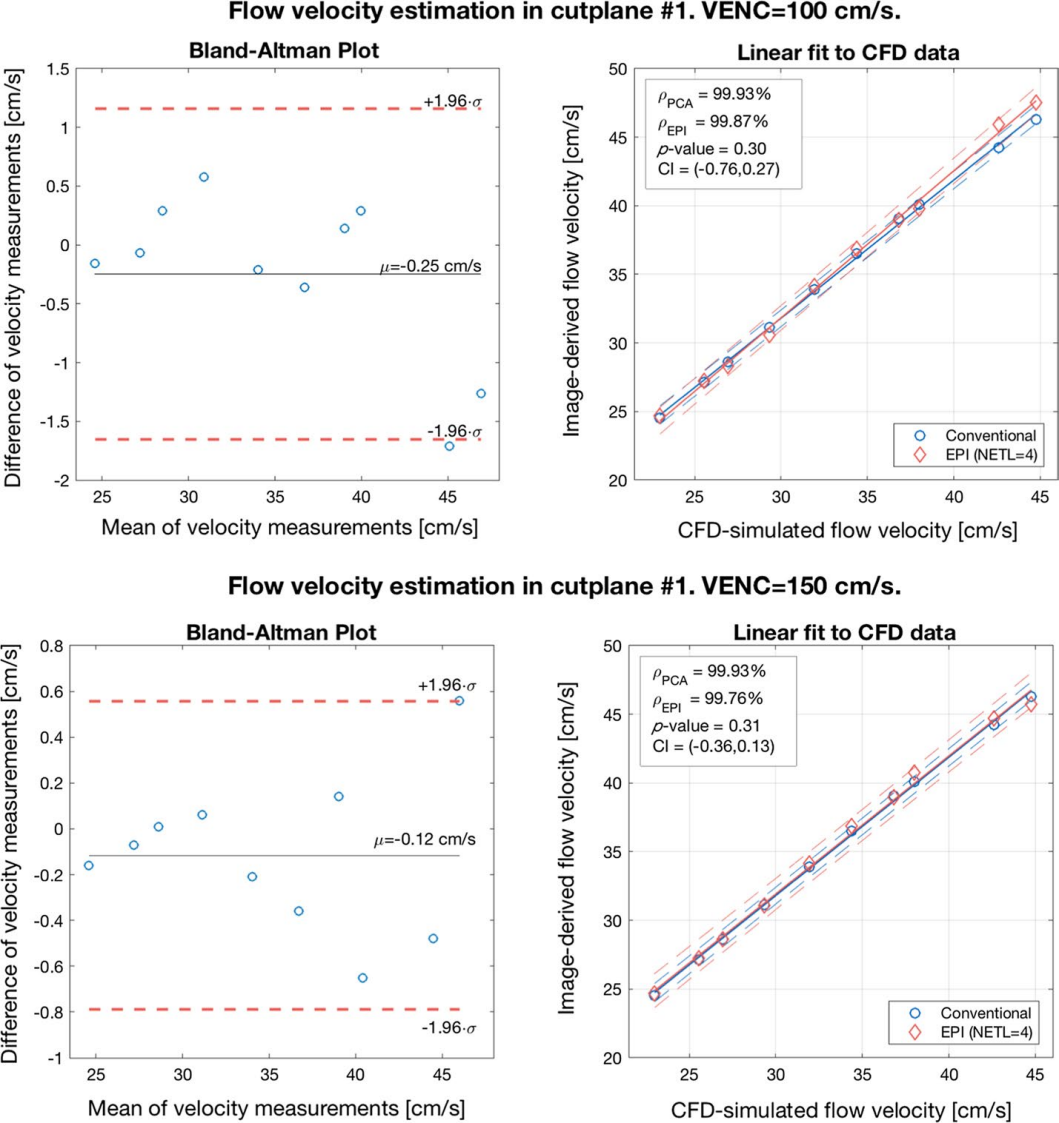
Bland–Altman plots for conventional and EPI-based velocity measurements and linear fitting of the image-derived estimates to reference velocities simulated in Comsol Multiphysics

In this part of experiments, we simulated 2-D PCA imaging for two selected cross-sections of the renal artery in the CALIX model. As marked in Fig. 7, the first cross-section was chosen in the entry region of the artery, whereas the second one was located after the artery split into sub-trees. As noted before, the velocity encoding gradients were switched on only in the slice-selection direction. The measurements were repeated for ten various inlet velocities, two VENC parameter values (100 and 150 cm/s) and three echo train lengths, as reported in Table 1. In order to enable visual evaluation of the results, the PCA-based velocity maps are accompanied by the corresponding reference (ground-truth) plots created in the flow simulation software (Figs. 10, 11, 12). For quantitative comparison of conventional and EPI readouts, we first averaged velocity estimates over the corresponding cross-sectional areas. Then, agreement between two measurement methods was evaluated using the Bland–Altman plots (shown in Fig. 13, left-hand side), separately for different VENC and EPI acceleration factors. On the other hand, the measurement errors are calculated against the ground truth values. In the latter case, we performed linear fitting of the image-derived velocities to the CFD-simulated quantities and calculated correlation coefficients between thereof (Fig. 13, right-hand side). We also conducted a paired *t*-test in order to verify whether the observed differences in the measurement errors are significant.

The analysis of the obtained results reveals high level of agreement between conventional PCA and the EPI-based variant with NETL = 4. The absolute mean difference in velocity estimates does not exceed 0.25 cm/s for VENC = 100 and 0.12 cm/s for VENC = 150 cm/s. Also, both methods appear equally accurate in approximation of the true flow velocity values. Correlation coefficients calculated between series of cross-sectional velocity averages, for both conventional and EPI schemes, surpass 99%. Consequently, at the confidence levels greater than 90%, the *t* test disallows rejecting the null hypothesis of there being no difference between the corresponding measurement errors (*p *≥ 0.3).

When analyzing the image-derived velocity maps with respect to the increasing value of blood flow speed, it becomes apparent that for conventional PCA, the shape of a vessel cross-section remains unaltered. In case of the EPI readout and the higher simulated flow velocities, the imaging artifacts are observed and manifest in signal blurring in the phase-encoding direction. This effect results in larger observed velocity measurement errors what can be noticed e.g. in Figs. 11 and 12 (bottom rows) or in Fig. 13 (Bland–Altman plots) in relation to peak velocities or cross-section averages, respectively. In the latter case, the biases between conventional PCA and EPI-based measurements for the higher speed rates reaches the limits of the estimated 95%-confidence interval.

**Fig. 14:**
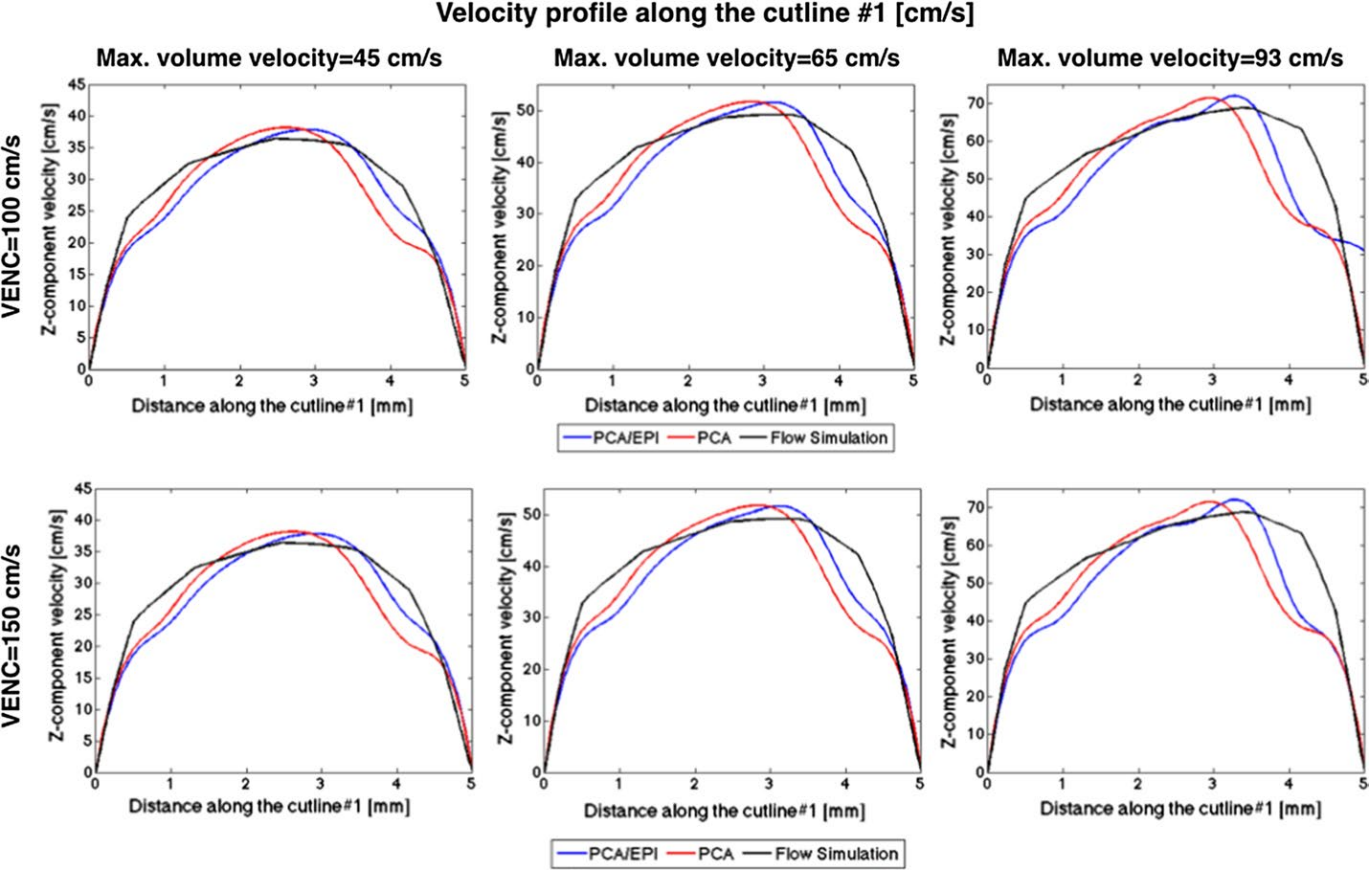
Examples of velocity profiles along the cutline in cross-section #1

The velocity profiles drawn for cutline #1 also exhibit high level of similarity between conventional PCA, PCA with EPI and the reference curve plotted from the flow simulation data, as shown on the example plots depicted in Fig. 14. The peak velocities (in the middle of the artery) estimated from the image data are slightly higher than the reference values. On the other hand, velocities measured at pixels lying further from the vessel axis are underestimated. The largest observed differences reach the value of 24 cm/s. However the overall shape of the corresponding curves are congruent. It is especially apparent for the highest flow velocity (93 cm/s) as well as for the cutline #2, where the velocity profile deviates from ideally symmetric parabolic curve.

**Fig. 15:**
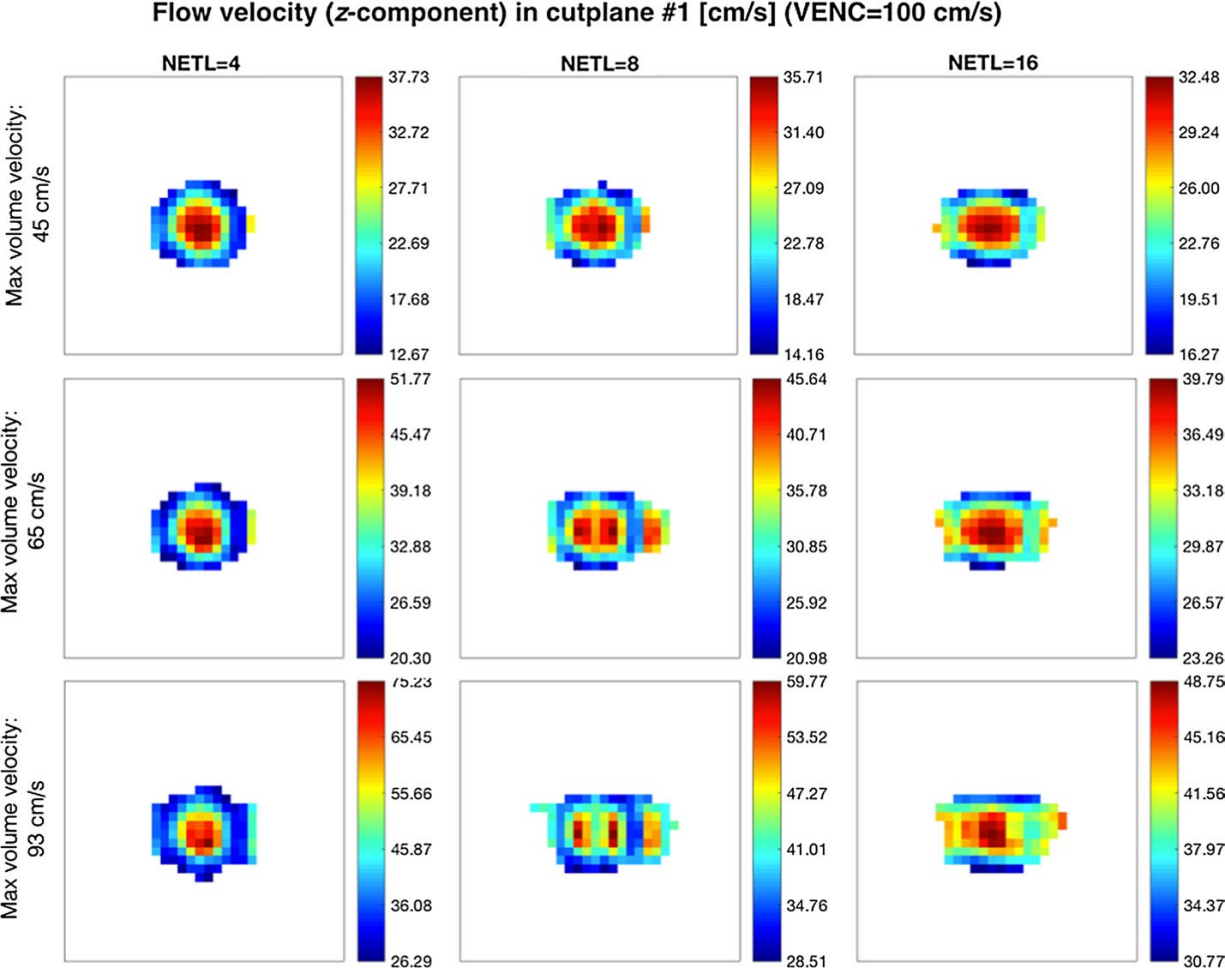
Examples of the magnitude velocity maps for cutplane #1 with respect to various echo train lengths and flow velocity rates

**Fig. 16:**
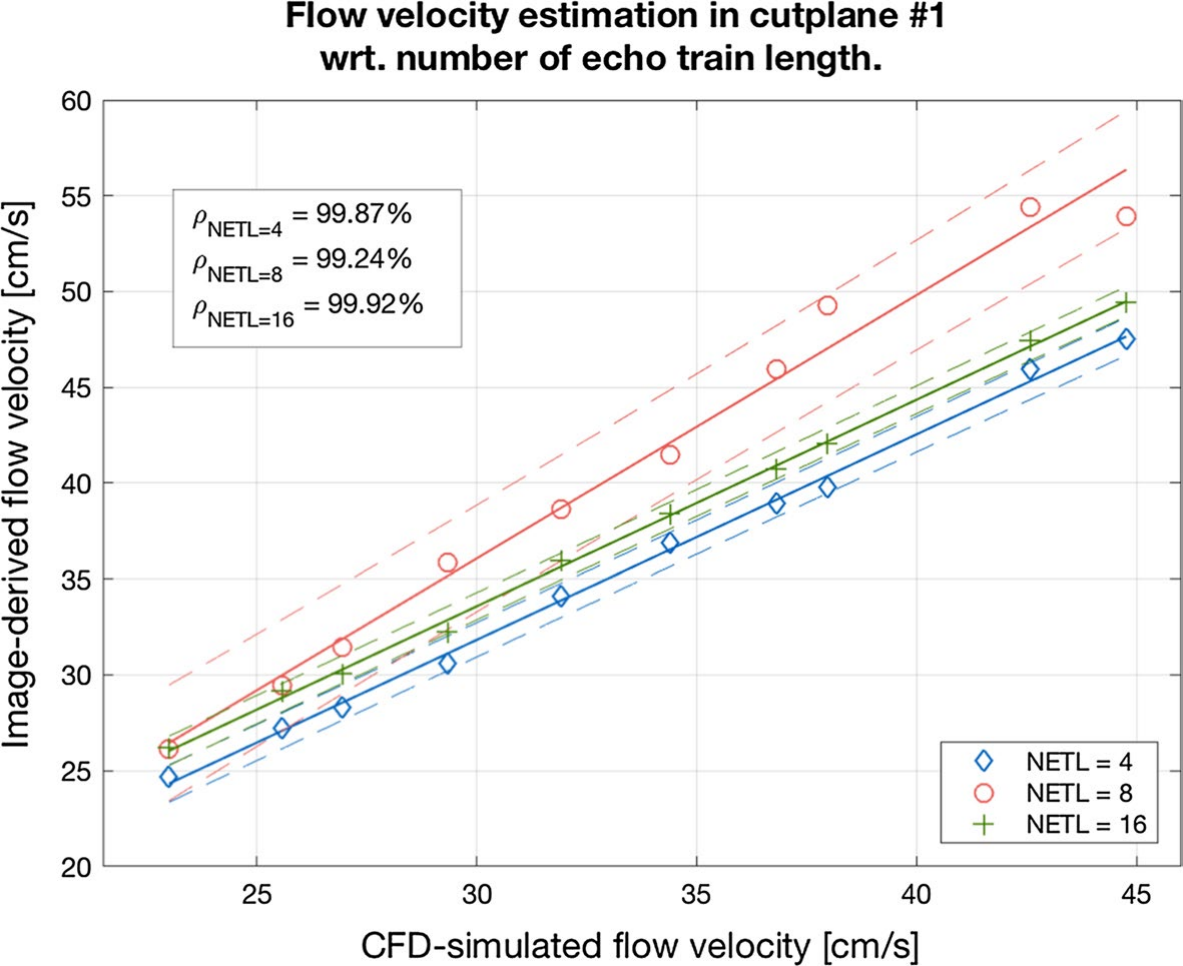
Linear fitting of the image-derived velocities estimated based on PCA images synthesized using different EPI acceleration factors to reference speed rates simulated in Comsol Multiphysics

The EPI-related phase artifacts become even more pronounced with increased number of echos (Fig. 15). Especially the fastest-flowing spins are spatially miscoded and it results in false signal mapping in reconstructed images. This is less noticeable for NETL = 4 since the shifted locations have much smaller intensity relative to the actual signal position and they are easily masked out from the phase images by the magnitude data. Moreover, the peak velocity values become remarkably underestimated for the higher EPI acceleration ratios. Depending on the velocity measurement level, this underestimation ranges from 14 to 34%. Apparently, the cross-sectional averages still allow accurate approximations of the true values, as shown on the linear fitting diagram in Fig. 16.

## Discussion

The presented study illustrates usage of the designed MR angiography simulator in application to quantitative validation of image-based measurement of blood velocity in renal arteries. The proposed experimental framework involved development of one simulated and eight patient-specific phantoms of the kidney vasculature, so as to mimic realistic flow conditions characteristic for renal hemodynamics. Our implementation of the MR imaging system enables simulation of the phase contrast angiography sequence, optionally with the usage of echo-planar readouts. We thus presented, that the proposed approach facilitates assessment of image-based flow velocity rating through direct comparison of the measured values with ground-truth data, not available in case of real examinations. An attractive feature of this strategy is the ability to study the impact of various scanning options, such as flip angle, resolution or the value of aliasing velocity, in isolation from other factors.

Based on the obtained results, the PCA method emerges as a feasible technique for diagnosing the kidneys. The mean measurement error in case of a 3D sequence reaches the level of 12.8% in the worst case but it can be reduced by adjusting the acquisition parameters, such as flip angle or slice spacing. In 2D, the observed discrepancies between the estimated and reference values are even smaller. However, high level of agreement has been achieved due to maximization of the through-plane flow. Therefore, under real scanning conditions, similar accuracy could be achieved if the orientation of oblique slice selection axis is properly determined. From the studied imaging parameters, the influence of the flip angle on the measurement accuracy occurred to be the most remarkable observation. However, the effect of in-plane resolution and slice spacing, although self-evident, was objectively quantified. All these effects, along with the other factors not studied here but also adjustable by the simulation framework (such as e.g. repetition and echo times or width of the acquisition window), should be considered when designing and optimizing specific PCA sequences.

Although we put much effort in the development of realistic renal arterial system phantoms, the complexity of the underlying hemodynamics was not fully reproduced in the finally designed models. The assumption of the laminar flow and blood incompressibility simplifies the computations, but does not reflect the true flow regime. Moreover, under real imaging condition the velocity estimation errors might be larger than calculated in this simulation study as additional examination factors would come into play. These include motion-related and susceptibility artifacts or partial volume effects. Therefore, the designed simulator and the accuracy of the achieved results should be further validated against the PCA sequence run on a real MRI scanner. Hence, the achieved results must be evaluated in light of the presumed simplifications.

Future research is planned into further extension of the designed MRA simulator. It includes implementation of the EPI technique in 3D, as well as other acceleration techniques, such as undersampling and projection reconstruction. We also plan to account for more complex flow phenomena, such as turbulent and pulsatile flow. These improvements will enable more in-depth investigation of the PCA sequence behavior and its applicability in diagnosing renal performance.

## Conclusions

Firstly, it must be underlined, that the proposed approach to modeling kidney vascular trees leads to constructing realistic renal arterial models, tractable by flow and MR imaging simulation software. Extraction of proper geometrical descriptions of vessel walls and usage of Comsol’s built-in modeling tools facilitates design of the arterial trees which are free from common surface- and edge-related defects otherwise generated by automatic tools.

Secondly, the presented simulation framework enables quantification of the measurement error committed by the PCA imaging sequences. The image-derived velocities can be compared against ground-truth data available from blood flow simulation on a voxel by voxel basis. The mean velocity measurement error for the reconstructed renal arterial trees range from 1.5 to 12.8% of the VENC value, depending on image resolution and flip angle.

Moreover, the designed simulator enables quantitative validation of an accelerated PCA sequence based on echo-planar imaging against conventional readout. No statistically significant difference was observed between velocity measurements obtained using echo-planar imaging with NETL = 4 and conventional sequence. Finally, for higher acceleration factors, i.e. NETL = 8 or 16, the peak velocity values in selected image slices were considerably underestimated by 14–34%.

## Abbreviations

DUS: Doppler ultra-sound
CT: computed tomography
EPI: echo-planar imaging
FA: flip angle
ITK: image processing toolkit
MRA: magnetic resonance angiography
MRI: magnetic resonance imaging
NETL: number of echos/echo train length
PCA: phase-contrast angiography
PE: phase encoding axis
RA: renal artery
RAS: renal artery stenosis
RO: read-out axis
SS: slice selection/slab selection axis
TE: echo time
TR: repetition time
VENC: aliasing velocity/velocity encoding gradient
VTK: visualization toolkit

## References

[1] Li S, Zöllner FG, Merrem AD, Peng Y, Rørvik J, Lundervold A, Schad LR (2012). Wavelet-based segmentation of renal compartments in DCE-MRI of human kidney: initial results in patients and healthy volunteers. Comput Med Imaging Graph.

[2] Zöllner FG, Svarstad E, Munthe-Kaas AZ, Schad LR, Lundervold A, Rørvik J (2012). Assessment of kidney volumes from MRI: acquisition and segmentation techniques. Am J Radiol.

[3] Schoenberg SO, Knopp MV, Bock M, Kallinowski F, Just A, Essig M, Hawighorst H, Schad L, van Kaick G (1997). Renal artery stenosis: grading of hemodynamic changes with cine phase-contrast MR blood flow measurements. Radiology.

[4] François CJ, Lum DP, Johnson KM, Landgraf BR, Bley TA, Reeder SB, Schiebler ML, Grist TM, Wieben O (2011). Renal arteries: isotropic, high-spatial resolution, unenhanced MR angiography with three-dimensional radial phase contrast. Radiology.

[5] Bock M, Schoenberg SO, Schad LR, Knopp MV, Essig M, van Kaick G (1998). Interleaved gradient echo planer (IGEPI) and phase contrast CINE-PC flow measurements in the renal artery. J Magn Reson Imaging.

[6] Granata A, Fiorini F, Andrulli S (2009). Doppler ultrasound and renal artery stenosis: an overview. J Ultrasound.

[7] Ng YY, Shen SH, Wang H-K, Tseng HS, Lee RC, Wu SC (2010). Magnetic resonance angiography and Doppler scanning for detecting atherosclerotic renal artery stenosis. J Chin Med Assoc.

[8] Clark DJ, Lessio S, O’Donoghue M, Schainfeld R, Rosenfield K (2004). Safety and utility of intravascular ultrasound-guided carotid artery stenting. Cathet Cardiovasc Interv.

[9] Su S, Hu Z, Lin Q, Kongto Hau W, Gao Z, Zhang H (2017). An artificial neural network method for lumen and media-adventitia border detection in IVUS. Comput Med Imaging Graph.

[10] Zhang HL, Sos TA, Winchester PA, Gao J, Prince MR (2009). Renal artery stenosis: imaging options, pitfalls, and concerns. Prog Cardiovasc Dis.

[11] Klepaczko A, Szczypiński P, Strzelecki M, Materka A (2015). Numerical modeling of MR angiography for quantitative validation of image-driven assessment of carotid stenosis. IEEE Trans Nucl Sci.

[12] Richter CS, Krestin GP, Eichenberger AC, Schöpke W, Fuchs WA (1993). Assessment of renal artery stenosis by phase-contrast magnetic resonance angiography. Eur Radiol.

[13] de Haan MW, van Engelshoven JMA, Houben AJHM, Kaandorp DW, Kessels AGH, Kroon AA, de Leeuw PW (2003). Phase-contrast magnetic resonance flow quantification in renal arteries. Comparison with ^133^Xenon washout measurements. Hypertension.

[14] Marshall I (2010). Computational simulations and experimental studies of 3D phase-contrast imaging of fluid flow in carotid bifurcations geometries. J Magn Reson Imaging.

[15] Petersson S, Dyerfeldt P, Gardhagen R, Karlson M, Ebbers T (2010). Simulation of phase contrast MRI of turbulent flow. Magn Reson Med.

[16] Szczypiński P. *VesselKnife*. http://eletel.p.lodz.pl/pms/SoftwareVesselKnife.html. Accessed June 2017.

[17] Kwan RS, Evans A, Pike G (1999). MRI simulation-based evaluation of image-processing and classification methods. IEEE Trans on Med Imaging.

[18] Jochimsen TH, Von Mengershausen M (2004). ODIN-Object-oriented development interface for NMR. J Magn Reson.

[19] Yoder DA, Zhao Y, Paschal CB, Fitzpatric JM (2004). MRI simulator with object-specific field map calculations. Magn Reson Imaging.

[20] Benoit-Cattin H, Collewet G, Belaroussi B, Saint-Jalmes H, Odet C (2005). The SIMRI project: a versatile and interactive MRI simulator. J Magn Reson Imaging.

[21] Drobnjak I, Pell G, Jenkinson M (2010). Simulating the effects of time-varying magnetic fields with a realistic simulated scanner. Magn Reson Imaging.

[22] Stoecker T, Vahedipour K, Pugfelder D, Shah NJ (2010). High-performance computing MRI simulations. Magn Reson Med.

[23] Fortin A, Salmon S, Baruthio J, Delbany M, Durand E (2018). Flow MRI simulation in complex 3D geometries: application to the cerebral venous network. Magn Reson Med.

[24] Cao Z, Oh S, Sica CT, McGarrity JM, Horan T, Luo W, Collins CM (2014). “Bloch-based MRI system simulator considering realistic electromagnetic fields for calculation of signal, noise, and specific absorption rate. Magn Reson Med.

[25] Liu F, Velikina JV, Block WF, Kijowski R, Samsonov AA (2017). Fast realistic MRI simulations based on generalized multi-pool exchange tissue model. IEEE Trans Med Imaging.

[26] Marshall I (1999). Simulation of in-plane flow imaging. Concepts Magn Reson.

[27] Li L, Doyle M, Rayarao G, Kortright E, Ito Y, Anayiotos A (2007). Numerical simulation of in vitro pulsatile flow and its study using FRISK, a rapid phase contrast technique. J Magn Reson Imaging.

[28] Klepaczko A, Szczypiński P, Dwojakowski G, Strzelecki M, Materka A (2014). Computer simulation of magnetic resonance angiography imaging: model description and validation. PLoS ONE.

[29] COMSOL Multiphysics Version 5.2a. CFD module user’s guide. Comsol AB, Stockholm, Sweden.B.

[30] Fedorov A, Beichel R, Kalpathy-Cramer J, Finet J, Fillion-Robin JC, Pujol S, Bauer C, Jennings D, Fennessy FM, Sonka M, Buatti J, Aylward SR, Miller JV, Pieper S, Kikinis R (2012). 3D Slicer as an image computing platform for the quantitative imaging network. Magn Reson Imaging..

[31] Frangi AF, Niessen WJ, Vincken KL, Viergever MA, Wells WM, Colchester A, Delp SL (1998). Multiscale vessel enhancement filtering. Medical image computing and computer-assisted intervention, MICCAI’98.

[32] Klepaczko A, Szczypiński P, Deistung A, Reichenbach J, Materka A (2016). Simulation of MR angiography imaging for validation of cerebral arteries segmentation algorithms. Comput Methods Programs Biomed.

[33] Johnson HJ, McCormick MM, Ibanez L (2015). The ITK software guide: design and functionality.

[34] Schroeder W, Martin K, Lorensen B (2006). The visualization toolkit.

[35] Wong KKL, Wang D, Ko JKL, Mazumdar J, Le TT, Ghista D (2017). Computational medical imaging and hemodynamics framework for functional analysis and assessment of cardiovascular structures. Biomed Eng Online.

[36] Kamiya A, Togawa T (1972). Optmial branching structure of the vascular tree. Bull Math Biophys.

[37] Bezy-Wendling J, Bruno A (1999). A 3D dynamic model of vascular trees. J Biol Syst.

[38] Rosset A, Spadola L, Ratib O (2004). OsiriX: an open-source software for navigating in multidimensional DICOM images. J Digit Imaging.

[39] Liang ZP, Lauterbur PC (2000). Principles of magnetic resonance imaging: a signal processing perspective.

[40] Basha TA, Akçakaya M, Goddu B, Berg S, Nezafat R (2015). Accelerated three-dimensional cine phase contrast imaging using randomly undersampled echo planar imaging with compressed sensing reconstruction. NMR Biomed.

[41] Bernstein MA, King KF, Zhou XJ (2004). Handbook of MRI pulse sequences.

[42] Greene ER, Venters MD, Avasthi PS, Conn RL, Jahnke RW (1981). Noninvasive characterization of renal artery blood flow. Kidney Int.

[43] Xu C, Xiong H, Gao Z, Liu X, Zhang H, Zhang Y, Du X, Wu W, Liu G, Li S (2017). Beat-to-beat blood pressure and two-dimensional (axial and radial) Motion of the carotid artery wall: physiological evaluation of arterial stiffness. Sci Rep.

